# Genetic Polymorphisms of Estrogen Receptors α and β and the Risk of Developing Prostate Cancer

**DOI:** 10.1371/journal.pone.0006523

**Published:** 2009-08-05

**Authors:** Young Kwang Chae, Han-Yao Huang, Paul Strickland, Sandra C. Hoffman, Kathy Helzlsouer

**Affiliations:** 1 Department of Medicine, Albert Einstein Medical Center, Philadelphia, Pennsylvania; United States of America; 2 Department of Epidemiology, Bloomberg School of Public Health, Johns Hopkins University, Baltimore, Maryland, United States of America; 3 Outcomes Research and Management, Merck & Co., West Point, Pennsylvania, United States of America; 4 The Prevention and Research Center, Mercy Medical Center, Baltimore, Maryland, United States of America; Brunel University, United Kingdom

## Abstract

Estrogen may be involved in the development of prostate cancer. The association between genetic polymorphisms of estrogen receptors α (ESR1) and β (ESR2) and prostate cancer risk was examined in a nested case-control study in Washington County, Maryland. Incident prostate cancer cases (n = 269) were matched to one or two controls (n = 440) by age, sex, race, and date of blood donation. Associations between estrogen receptor genotypes or dietary intake and the development of prostate cancer were examined in conditional logistic regression models. Results from this study showed that six single base-pair polymorphisms (SNPs) of ESR1 (rs1801132, rs2077647, rs746432, rs2273206, rs851982, rs2228480) and four SNPs of ESR2 (rs4986938, rs928554, rs8018687, rs number not available for ESR2 5696 bp 3′ of STP A>G) were not significantly associated with prostate cancer risk, either by allelic or genotypic frequencies. However, an interactive association with BMI was observed in the relationship between prostate cancer risk and genotypes of ESR2 38 bp 3′ of STP G>A (rs4986938) (p = 0.031). An interaction between intake level of phytoestrogen and genotypes of ESR1 Ex1-192G>C (rs746432) and between intake level of phytoestrogen and genotypes of ESR1 Ex8+229G>A (rs2228480) and risk of prostate cancer was observed (p = 0.0009 and p = 0.044, respectively). In conclusion, selected genetic polymorphisms of ESR1 and ESR2, overall, were not associated with prostate cancer risk. However, a variation in risk by BMI and phytoestrogen intake was implicated.

## Introduction

The development of prostate cancer may be hormone-dependent [1], [2]. For example, androgens are important for normal and hyperplastic prostate growth. Both testosterone and dihydrotestosterone (DHT) induced prostatic adenocarcinoma in rat models [3]. Anti-androgen therapy or orchiectomy has been used to treat metastatic prostate cancer [4]. Estrogen as well as androgen may play an important role in the carcinogenesis of prostate cells [5]. It was initially thought that estrogens mediate their action through estrogen receptor α (ESR1). Estrogen receptor β (ESR2) was later identified to be involved in the process [6], [7]. Although these two receptors share 47% structural similarities, they can be differentiated by their physiological properties [6], [8]. While ESR1 is mainly localized in the prostatic stroma [9], ESR2 is mostly located in the prostatic epithelium [10]. ESR1 plays an essential role in prostate development [11] and is found to be related to estrogen-induced prostatic squamous metaplasia [12]. Also, its expression at tumor level negatively correlates with prostate cancer survival [13]. On the other hand, ESR2 is thought to be an important regulator of prostate function [14], especially as a potential “brake” to androgen-driven proliferation [13].

Several studies have suggested a relationship between these two receptors and prostate cancer [15]–[21]. For example, exposure to high levels of estrogen in uteri may lead to smaller adult prostates that are poorly sensitive to androgen for the development of hyperplasia, inflammation and dysplasia [16]. This genetic imprinting is found to be mediated by ESR1 [16]. Other studies have consistently found that ESR2 is expressed in metastatic prostate cancer cells [17]–[19]. Accordingly, phytoestrogens, chemicals produced by plants that mimic estrogen effects, may act like ESR2 agonist. It has been hypothesized that phytoestrogen may be protective against prostate cancer [20], [21].

Genetic polymorphisms of the ESR1 and ESR2 have been reported to be associated with prostate cancer risk [22]–[34]. However, no biological functional studies have been published to support the epidemiologic findings, and analyses of gene-environment interactions were rarely performed. Identifying the environmental factors that may modify the relationship between genetic polymorphisms and disease risk may provide a clue to possible functions of the genetic polymorphisms or to the locations of functional SNPs. Body mass index (BMI) may be a proxy indicator for the relative amount of body fat, which is a major source of estrogen production in men [35]. Availability of estrogen in the body may affect the sensitivity of estrogen receptors, possibly leading to a different risk profile for prostate carcinogenesis. On the other hand, phytoestrogen, rich in legumes, may stimulate or modulate estrogen receptors, particularly for ESR2 [36].

A nested case-control study was conducted to examine the associations between selective polymorphisms of ESR 1, ESR2 genes and the risk of developing prostate cancer in a community-based cohort in Washington County, Maryland. Exploratory analyses examined how the ESR1 and ESR2 SNPs of interest modified the association between BMI/phytoestrogen and prostate cancer risk and also how BMI and phytoestrogen altered the association between the ESR1 and ESR2 SNPs of interest and prostate cancer risk.

## Materials and Methods

### Study population

CLUE II was a slogan of the second research campaign, “Give us a CLUE to cancer and Heart Disease”, conducted in Washington County, Maryland in 1989. The participants were 10,456 men and 14,625 women (total of 25,081). Approximately 30% of the Washington County adult population participated. Mobile office trailers were utilized to collect specimen. 20 ml of blood from each participant was put into 20 ml vacutainer. In addition to plasma aliquots, white blood cells and a sample with a vitamin C preservative were stored at −70°C. Buffy coats from the samples were used as a source of the genotyping analysis.

Study participants provided data on education, cigarette smoking (never, former, current), height, weight, medication use, and vitamin use in the year prior to questionnaire administration. They also filled out a brief food frequency questionnaire [37] that included questions about the serving size and frequency of intake of 61 food items. The annual loss-to-follow-up in the cohort was less than 1 percent.

A written informed consent to the participation in the research campaign was obtained from each participant at the time of blood donation. This study was approved by the institutional review board of the Johns Hopkins Bloomberg School of Public Health.

### Case ascertainment and control selection

Prostate cancer incident cases (International Classification of Diseases, 9th revision, code 185) were identified through linkage to the Washington County Cancer Registry and since 1992, also to the Maryland State Cancer Registry. All cases were confirmed pathologically (n = 269). Stages and grades were described according to American Joint Committee on Cancer/Tumor Node Metastasis (TNM) system and Gleason's Score system, respectively.

Cases were defined as participants who developed primary prostate cancer during the follow up period from 1989 to 2002. Each prostate cancer case was individually matched with one or two controls on age (±1 year), gender, ethnicity, and date of blood donation. One-to-one and one-to-two matching was done for 36% and 64%, respectively, of the cases. Each control was selected from the CLUE II cohort, not known to have cancer except for basal or squamous cell skin cancer and not known to have died, at the time when the corresponding case was diagnosed.

### Genotyping

Heparinized blood samples were centrifuged at 1500 g for 30 minutes at room temperature within 6 hours of collection. Then they were separated into plasma, buffy coat, and red blood cells and were frozen at −70°C within 24 hours of collection. Genetic polymorphisms of estrogen receptor α (ESR1) and estrogen receptor β (ESR2) were determined on DNA samples extracted from the preserved participants' buffy coat specimens. The buffy coat remained frozen till DNA extraction was done. The alkaline lysis method was used for the DNA extraction procedure. All genotypings were carried out using TaqMan® assays (Applied Biosystems, Foster city, CA, USA). Laboratory researchers handling samples were masked to disease status.

Candidate single nucleotide polymorphisms (SNPs) were chosen based on the following criteria: (a) the allele frequency of over five percent in published literature or databases, recommended by the National Cancer Institute [38], (b) validated allele substitutions, and/or (c) functional changes linked with allele substitution reported in the literature.

For describing SNP sequence variations, we adapted the recommendation from a Nomenclature Working Group [39]. Among the ESR1 SNPs, four SNPs including Ex4-122C>G, Ex1+392T>C, Ex1-192G>C, and Ex8+229G>A were in the coding region. The rest of the ESR1 SNPs were either in the non-coding region prior to ATG translation initiating codon (-104062C>T), or in the intron 6 region (IVS6+52G>T). All of the ESR2 SNPs were in the non-coding region after the translation terminating codon (38 bp 3′ of STP G>A, 5659 bp 3′ of STP A>G, 5696 bp 3′ of STP A>G, 5772 bp 3′ of STP A>G). Ten selected SNPs were genotyped in both cases and controls. Among cases, 13.1% (35/269) were missing any one genotype and among controls, 10.5% (46/440). Average missing rate for any one genotype was around 18%.(I have erased the accuracy part.)

### Statistical Analysis

Baseline characteristics between cases and controls were compared by conditional logistic regression models for categorical variables (Table 1). Based on self-reported height and weight at the time of blood donation, we calculated BMI as kilogram per square meter. Family history was identified by self report on the prostate cancer history of grandfathers, fathers and brothers. Dietary intake of fat, energy, phytoestrogen, and calcium was estimated by summing the product of the frequency of consumption of each food, the reported serving size, and the energy or nutrient content per serving. Total phytoestrogen intake was computed based on legume consumption. Isoflavone (phytoestrogen) contents were estimated for beans (pinto, lima, kidney, and other beans, possibly including soy), peas, and peanuts using USDA-Iowa State University Database on the Isoflavone Content of Foods, 1999 [40]. All quartile cut-offs used were based on the data in the control group. High/low phytoestrogen intake cut-off was the median level of intake in the control group. To deal with missing data for dietary (average of 30% missing) in calculating the total isoflavone intake, imputation analysis was performed, inserting zero or median in place of missing data, both of which did not affect the overall results.

**Table 1:** Characteristics of prostatic cancer cases and matched controls in the CLUE 2 cohort, 1989.

Characteristics	case subjects (n = 269)	control subjects (n = 440)	matched odds ratio	95% confidence interval
**Age, y, mean(SE)**	64.1( 9.0)	64.7(8.7)		
**Race, Black, n, (%)**	6(2.23)	11(2.50)		
**Education level, y, %**				
** <12**	27.1	34.1	1.00	
** = 12**	42.8	37.3	1.38	0.94, 2.02
** >12**	30.1	28.6	1.25	0.84,1.84
**Cigarette smoking, %**				
** never**	39.8	39.3	1.00	
** former**	51.7	50.2	1.02	1.75,1.41
** current**	8.5	10.5	0.83	0.44,1.40
**BMI at baseline, kg/m^2^, %**				
** <24.9**	33.1	30.2	1	
** 25–29.9**	52.4	55.9	0.84	0.59,1.19
** > = 30**	14.5	13.9	0.95	0.57,1.57
**BMI at age 21, kg/m^2^, %**				
** <24.9**	80.9	78.2	1	
** 25–29.9**	16.1	19.8	0.79	0.53,1.18
** > = 30**	3.0	2.1	1.19	0.42,3.34
**Vitamin supplement use, %**				
** multivitamin regular use** *	23.6	20.2	1.11	0.71,1.73
** ever vitamin C use** †	37.6	34.6	1.04	0.75,1.46
** ever vitamin D use** †	25.2	21.6	1.17	0.81,1.69
** ever vitamin E use** †	34.8	33.1	1.01	0.72,1.41
**Family history** ‡ **, %**				
** no**	58.7	63.4	1.00	
** yes**	8.6	5.5	1.69	0.82,3.48
** missing**	32.7	31.1		
**Dietary intake of fat, %**				
** 1st quartile** П	23.4	22.7	1.00	
** 2nd quartile** П	18.6	23.0	0.79	0.50,1.27
** 3rd quartile** П	24.5	22.7	0.93	0.58,1.50
** 4th quartile** П	26.4	23.0	1.08	0.68,1.70
** missing**	7.1	8.6		
**Dietary intake of total calories, %**				
** 1st quartile** П	20.1	22.7	1.00	
** 2nd quartile** П	25.3	23.0	1.22	0.77,1.93
** 3rd quartile** П	21.6	22.7	1.01	0.63,1.63
** 4th quartile** П	26.0	23.0	1.25	0.78,1.20
** missing**	7.1	8.6		
**Dietary intake of calcium, %**				
** 1st quartile** П	20.1	22.7	1.00	
** 2nd quartile** П	24.9	23.0	1.11	0.70,1.78
** 3rd quartile** П	26.0	22.7	1.22	0.78,1.92
** 3rd quartile** П	21.9	23.0	1.08	0.66,1.75
** missing**	7.1	8.6		
**Dietary intake of phytoestrogen, %**				
** 1st quartile** П	19.7	17.5	1.00	
** 2nd quartile** П	17.8	17.3	0.77	0.43,1.39
** 3rd quartile** П	13.4	17.3	0.58	0.31,1.09
** 4th quartile** П	21.2	17.3	0.97	0.55,1.71
** missing**	27.9	30.7		
**Stage of disease at diagnosis, %**				
**0**	1.1			
**1**	14.9			
**2**	32.0			
**3**	18.2			
**4**	3.7			
**missing**	30.1			
**Grade of disease at diagnosis** § **, %**				
**1**	7.8			
**2**	64.7			
**3**	7.4			
**missing**	20.1			
**Case/control, %**				
** one/one**	36.0			
** one/two**	64.0			

*regular users compared with nonusers and non-regular users among responder of the questionnaire.

† ever-users compared with nonusers among responders of the questionnaire.

‡ prostate cancer of grandfather, father, and brothers.

§ Grade: 1(Gleason score 2–4, well differentiated), 2(Gleason score 5–7, moderately differentiated), 3(Gleason score 8–10, poorly differentiated).

П All quartiles are derived from controls: 1) total caloric intake (n = 652) quartile cut off: 1107.8, 1438.4, 1861.8 Cal/d. 2) fat intake (n = 652) quartile cutoff: 42.1, 59.0, 78.2 Cal/d. 3) calcium intake (n = 652) quartile cutoff: 424.0, 616.4, 878.7 mg/d 4) phytoestrogen intake (n = 499) quartile cut off: 1.35, 2.71, 4.14 mg/mo.

Association between estrogen receptor genotypes and the development of prostate cancer was examined in conditional logistic regression analyses. Odds ratios and the corresponding 95% confidence intervals were derived from three different genetic effect models, including dominant, recessive and additive models. Additionally, analyses were stratified by cancer stage and grade.

Gene-environment interaction associations of BMI or dietary phytoestrogen (isoflavone) intake on the relationship between prostate cancer risk and estrogen receptor gene genotypes were assessed by both stratification analyses and the likelihood ratio test (LRT) that compared the conditional logistic regression models with and without interaction terms. Associations between ESR1 and ESR2 genotypes and prostate cancer risk were evaluated in strata of three BMI categories and separately, strata of high/low phytoestrogen intake, by unconditional logistic regression with adjustment for age and race. The same method was used to assess the associations between BMI or dietary phytoestrogen intake and prostate cancer risk in strata of estrogen receptor genotypes, with adjustment for caloric intake in the analysis on phytoestrogen intake, age, and race. For BMI, test for trend was performed on the median values in each three categories. For genotypes, the additive model was assumed in the tests for trend. All p-values were derived from two-sided test and were considered to be statistically significant if less than 0.05. All statistical analyses were performed using STATA Statistical Software, 9.0 (Stata Corporation, College Station, TX, 2005).

## Results

Cases and controls were comparable with respect to age, race, education, history of cigarette smoking, vitamin use, and dietary intakes of fat, total calories, phytoestrogen, and calcium (Table 1). Among the 188 cases whose disease stages were determined, 129 had localized disease, defined as TNM stage 0, 1 or 2, and 59 had advanced disease, defined as TNM stage 3 or 4 (Table 1). Hardy-Weinberg equilibrium was tested for Caucasians in controls, who were 263 (94%) and 429 (89%) in each group. All the SNPs were in Hardy-Weinberg equilibrium except for ESR2 5659 bp 3′ of STP A>G among cases and ESR1 IVS6+52G>T, ESR1 -104062C>T, 5659 bp 3′ of STP A>G among controls (Table S1). Observed major allele frequencies of ESR SNPs from the CLUE cohort were compared with SNP500 or dbSNP data if available, separately for Caucasians and African Americans. Among the eight SNPs compared, no statistically significant difference was found with the exception of ESR1 Ex8+229G>A for Caucasians (Table S2).

Table 2 presents odd ratio estimates of prostate cancer risk for each genotype of estrogen receptor gene SNP. In dominant, recessive, and additive models, no statistically significant association was found between the SNPs and prostate cancer risk. In addition, no significant trend was found in the number of alleles with respect to prostate cancer risk. Of all ten SNPs, only ESR1 Ex4-122C>G was consistently associated with increased prostate cancer risk across all subgroups defined by stages and grades of the cancer (Table 2). However, none of the results were statistically significant. Among advanced prostate cancer cases, for ESR1, C allele in Ex1+392T>C was associated with a statistically significant decreased risk of prostate cancer. The T allele in IVS6+52G>T was associated with an increased risk of prostate cancer but the trend in risk with burden of T alleles was not statisticially significant. For ESR2, A allele in 38 bp 3′ of STP G>A and A allele in 5659 bp 3′ of STP A>G were associated with an increased risk of advanced prostate cancer but association were not statistically significant. (Table 2).

**Table 2:** The genotype frequencies of ESR1,2 polymorphisms and prostate cancer risk.

SNP	Frequency		Odds Ratio (95%CI)							
(dbSNP ID)	No. Cases (%)	No. Controls (%)	All Cases (269)	LocalizedStage§ (129)	Advanced Stage§ (59)	Low GradeП (134)	High GradeП (57)	Dominant Model	Recessive Model	Additive Model
**ESR1 Ex4-122C>G (rs1801132)**										
**C/C**	128 (47.6)	230 (52.3)	1.00	1.00	1.00	1.00	1.00	1.00	1.00	1.00
**C/G**	80 (29.7)	127 (28.9)	1.08 (0.75,1.55)	1.05 (0.61,1.80)	1.22 (0.58,2.57)	1.16 (0.76,1.78)	1.07 (0.22,5.11)	1.15 (0.81,1.62)		1.17 (0.89,1.56)
**G/G**	13 (4.8)	12(2.7)	1.73 (.78,3.85)	2.33 (0.81,6.67)	1.65 (0.39,6.92)	1.30 (0.47,3.58)	2.32 (0.37,14.34)		1.69 (0.77,3.75)	1.37 (0.79,2.43)
**missing**	48 (17.8)	71(16.1)								
**P_ trend_**			0.257	0.230	0.422	0.412	0.414			
**ESR1 Ex1+392T>C (rs2077647)**										
**T/T**	64 (23.79)	102 (23.2)	1.00	1.00	1.00	1.00	1.00	1.00	1.00	1.00
**T/C**	104 (38.7)	173 (39.3)	0.92 (0.60,1.41)	0.97 (0.52,1.81)	0.45 (0.18,1.14)	0.87 (0.53,1.44)	1.22 (0.23,6.42)	0.90 (0.61,1.34)		0.93 (0.74,1.19)
**C/C**	51 (18.96)	94 (21.36)	0.87 (0.54,1.41)	1.13 (0.58,2.24)	0.27 (0.09,0.80)	0.85 (0.48,1.50)	0.41 (0.06,2.59)		0.92 (0.61,1.38)	0.86 (0.55,1.42)
**missing**	50 (18.6)	71(16.1)								
**P_ trend_**			0.582	0.686	0.515	0.56	0.34			
**ESR1 Ex1-192G>C (rs746432)**										
**G/G**	177 (65.8)	301 (68.41)	1.00	1.00	1.00	1.00	1.00	1.00		
**G/C,C/C** *	37 (13.8)	69(15.7)	0.96 (0.60.1.55)	1.03 (0.55,1.94)	0.75 (0.20,2.76)	0.98 (0.54,1.79)	2.65 (0.49,14.28)	0.96 (0.60.1.55)		
**Missing**	55 (20.5)	70 (15.91)								
**P_ trend_**			0.872	0.915	0.663	0.959	0.257			
**ESR1 IVS6+52G>T (rs2273206)**										
**G/G**	171 (63.6)	299 (68.0)	1.00	1.00	1.00	1.00	1.00	1.00	1.00	1.00
**G/T**	48 (17.8)	72 (16.4)	1.19 (0.79,1.78)	1.00 (0.54,1.84)	1.63 (0.74,3.55)	0.87 (0.52,1.43)	1.73 (0.28,10.58)	1.21 (0.82,1.79)		1.19 (0.84,1.68)
**T/T**	5 (1.9)	8 (1.8)	1.41 (0.44,4.49)	1.00 (0.18,5.50)	5.58 (0.56,55.10)	1.32 (0.29,5.96)	2.00 (0.13,31.98)		1.35 (0.43,4.27)	1.42 (0.71,2.82)
**Missing**	45 (16.7)	61 (13.9)								
**P_ trend_**			0.326	1.000	0.064	0.770	0.451			
**ESR1 -104062C>T (rs851982)**										
**C/C**	144 (53.5)	252 (57.3)	1.00	1.00	1.00	1.00	1.00	1.00	1.00	1.00
**C/T**	53 (19.7)	77 (17.5)	1.09 (0.71,1.67)	0.77 (0.40,1.46)	1.97 (0.73,5.33)	0.98 (0.58,1.66)	0.91 (0.20,4.12)	1.06 (0.70,1.60)		1.03 (0.73,1.44)
**T/T**	7 (2.6)	13 (3.0)	0.90 (0.33,2.47)	0.67 (0.16,2.76)	0.57 (0.06,5.28)	1.59 (0.50,5.01)	.		0.88 (0.32,2.40)	1.06 (0.53,2.07)
**missing**	65 (24.2)	98 (22.3)								
**P_ trend_**			0.884	0.360	0.584	0.654	0.607			
**ESR1 Ex8+229G>A (rs2228480)**										
**G/G**	77 (28.6)	136 (30.9)	1.00	1.00	1.00	1.00	1.00	1.00	1.00	1.00
**G/A**	102 (37.9)	183 (41.6)	1.03 (0.70,1.53)	1.19 (0.69,2.07)	0.87 (0.38,2.03)	1.18 (0.73,1.89)	0.71 (0.15,3.38)	1.07 (0.74,1.54)		1.07 (0.84,1.38)
**A/A**	40 (14.9)	60 (13.6)	1.17 (0.70,1.96)	0.98 (0.47,2.03)	1.39 (0.41,4.65)	1.31 (0.70,2.46)	1.16 (0.13,9.99)		1.15 (0.72,1.83)	1.14 (0.71,1.90)
**Missing**	50 (18.6)	61(13.9)								
**P_ trend_**			0.579	0.905	0.726	0.358	0.926			
**ESR2 38 bp 3′ of STP (rs4986938) G>A**										
**G/G**	81 (30.1)	134 (30.5)	1.00	1.00	1.00	1.00	1.00	1.00	1.00	1.00
**G/A**	105 (39.0)	185 (42.1)	0.95 (0.64,1.41)	0.90 (0.52,1.58)	1.41 (0.57,3.46)	0.77 (0.48,1.22)	0.89 (0.17,4.69)	0.96 (0.66,1.39)		0.98 (0.76,1.27)
**A/A**	33 (12.3)	51 (11.6)	0.98 (0.57,1.70)	1.08 (0.48,2.46)	2.98 (0.88,10.09)	0.78(0.41,1.51)	2.41 (0.19,31.23)		1.01 (0.61,1.68)	0.96 (0.58,1.61)
**missing**	50 (18.6)	70 (15.9)								
**P_ trend_**			0.895	1.000	0.090	0.330	0.637			
**ESR2 5659 bp 3′ of STP A>G (rs928554)**										
**G/G**	28 (10.4)	58 (13.2)	1.00	1.00	1.00	1.00	1.00	1.00		1.00
**G/A**	117 (43.5,53.9)	193 (43.9)	1.21 (0.71,2.07)	1.17 (0.57,2.41)	2.35 (0.68,8.06)	0.95 (0.51,1.79)	1.57 (0.26,9.46)	1.19 (0.72,1.97)		1.05 (0.81,1.38)
**A/A**	67(24.9)	111 (25.2)	1.16 (0.66,2.03)	1.19 (0.53,2.65)	2.54 (0.75,8.60)	0.86 (0.45,1.66)	1.89 (0.24,14.87)		1.01 (0.68,1.49)	1.10 (0.66,1.90)
**Missing**	57(21.2)	78 (17.7)								
**P_ trend_**			0.700	0.691	0.189	0.627	0.561			
**ESR2 5696 bp 3′ of STP A>G (NA** §§ **)**										
**A/A**	181(67.3)	316 (71.8)	1.00	1.00	1.00	1.00	1.00	1.00		
**A/G,G/G** †	33(12.3)	53 (12.1)	1.04 (0.61,1.75)	1.04 (0.53,2.03)	0.82 (0.24,2.85)	1.02 (0.55,1.87)	0.75 (0.12,4.75)	1.04 (0.61,1.75)		
**Missing**	55(20.5)	71 (16.1)								
**P_ trend_**			0.894	0.910	0.756	0.959	0.758			
**ESR2 5772 bp 3′ of STP A>G (rs8018687)**										
**A/A**	191 (71.0)	324 (73.6)	1.00	1.00	1.00	1.00	1.00	1.00		
**A/G,G/G** ‡	31 (11.5)	50 (10.9)	1.07 (0.64,1.79)	1.16 (0.60,2.25)	1.05 (0.36,3.03)	1.13 (0.63,2.04)	.	1.07 (0.64,1.79)		
**Missing**	47 (17.5)	66 (15.0)								
**P_ trend_**			0.550	0.650	0.928	0.684				

* C/C: two cases and five controls in C/C genotype category.

† one case and two controls in G/G genotype category.

‡ three cases and two controls in G/G genotype category.

§ Localized = TNM stage 0,1,2; Advanced = TNM stage 3,4.

П Low Grade = Grade 1,2; High Grade = Grade 3 (Grade: 1(Gleason score 2–4, well differentiated), 2(Gleason score 5–7, moderately differentiated), 3(Gleason score 8–10, poorly differentiated).

§§ no data available.

Blanks cells denote calculations unable to be performed due to small sizes.

Among the the group with low intake of phytoestrogen, men who had the variant homozygote G/G genotype in ESR1 Ex4-122C>G had a 5-fold increase in the odds of developing prostate cancer when compared with wild type homozygote C/C genotype (P = 0.02, p-value for trend = 0.04) (Table 3). In contrast, men who had a variant homozygote C/C genotype in ESR1 Ex1+392T>C and G/C, C/C genotype in ESR1 Ex1-192G>C had a decrease in the odds of developing prostate cancer by 63% (P = 0.017, p-value for trend = 0.015) and 75% (P = 0.004) compared to wild type homozygote T/T and G/G genotype, respectively (Table 3).

**Table 3:** Estrogen receptor gene polymorphisms and prostate cancer risk according to BMI and habitual dietary intake of Phytoestrogen.

SNP	Odds Ratio (95%CI)					
	Overall	Phytoestrogen*		BMI†		
	(case/control)	Low (101/153)	High (93/152)	<25 (89/133)	25–30 (141/246)	(30 (39/61)
**ESR1 Ex4-122C>G**						
**C/C**	1.00	1.00	1.00	1.00	1.00	1.00
**C/G**	1.08 (0.75,1.55)	1.32 (0.72,2.42)	1.19 (0.66,2.14)	0.71 (.38,1.35)	1.40 (0.86,2.26)	2.08 (0.71,6.05)
**G/G**	1.73 (0.78,3.85)	**5.16 (1.24,21.51)**	1.85 (0.51,6.80)	2.48 (0.59,10.42)	1.68 (0.36,7.76)	1.95 (0.45,8.44)
**P_ trend_**		**0.041**	0.338	0.946	0.151	0.197
**ESR1 Ex1+392T>C**						
**T/T**	1.00	1.00	1.00	1.00	1.00	1.00
**T/C**	0.92 (0.60,1.41)	0.61 (0.32,1.18)	1.12 (0.56,2.25)	1.05 (0.51,2.17)	1.10 (0.64,1.89)	0.67 (0.23,1.91)
**C/C**	0.87 (0.54,1.41)	**0.37 (0.17,0.84)**	1.38 (0.63,3.06)	0.96 (0.44,2.08)	0.95 (0.49,1.85)	0.39 (0.09,1.03)
**P_ trend_**		**0.015**	0.421	0.914	0.924	0.196
**ESR1 Ex1-192G>C**						
**G/G**	1.00	1.00	1.00	1.00	1.00	1.00
**G/C,C/C**	0.96 (0.60.1.55)	**0.25 (0.10,0.64)**	1.32 (0.64,2.71)	0.65 (0.28,1.50)	1.16 (0.65,2.07)	0.76 (0.21,2.76)
**P_ trend_**		**0.004**	0.45	0.315	0.61	0.674
**ESR1 IVS6+52G>T**						
**G/G**	1.00	1.00	1.00	1.00	1.00	1.00
**G/T**	1.19 (0.79,1.78)	1.20 (0.57,2.51)	1.61 (0.84,3.07)	1.88 (0.85,4.16)	1.12 (0.64,1.98)	0.65 (0.22,1.94)
**T/T**	1.41 (0.44,4.49)	0.93 (0.16,5.47)	5.59 (0.37,84.23)	0.68	7.98 (0.73, 86.21)	‡NA
**P_ trend_**		0.755	0.07	0.408	0.24	0.239
**ESR1 -104062C>T**						
**C/C**	1.00	1.00	1.00	1.00	1.00	1.00
**C/T**	1.09 (0.71,1.67)	0.84 (0.43,1.66)	1.05 (0.50,2.18)	1.00 (0.49,2.03)	1.42 (0.82,2.48)	0.79 (0.24,2.57)
**T/T**	0.9 (0.33,2.47)	0.67 (0.11,3.87)	1.70 (0.41,7.09)	0.76 (0.17,3.33)	1.63 (0.42,6.31)	‡NA
**P_ trend_**		0.525	0.565	0.81	0.178	0.205
**ESR1 Ex8+229G>A**						
**G/G**	1.00	1.00	1.00	1.00	1.00	1.00
**G/A**	1.03(0.70,1.53)	1.06 (0.56,2.01)	0.58 (0.31,1.08)	0.87 (0.46,1.67)	1.24 (0.74,2.08)	0.71 (0.26,1.93)
**A/A**	1.17(0.70,1.96)	2.05 (0.92,4.58)	0.56 (0.24,1.29)	1.26 (0.54,2.92)	1.28 (0.65,2.53)	0.75 (0.18.3.15)
**P_ trend_**		0.115	0.101	0.146	0.418	0.585
**ESR2 38 bp 3′ of STP G>A**						
**G/G**	1.00	1.00	1.00	1.00	1.00	1.00
**G/A**	0.95 (0.64,1.41)	1.10 (0.59,2.06)	0.92 (0.50,1.68)	1.24 (0.65,2.39)	1.03 (0.62,1.71)	**0.28 (0.09,0.86)**
**A/A**	0.98 (0.57,1.70)	1.42 (0.60,3.37)	1.07 (0.46,2.51)	1.35 (0.55,3.33)	0.92 (0.44,1.90)	1.17 (0.29,4.74)
**P_ trend_**		0.455	0.986	0.455	0.875	0.443
**ESR2 5659 bp 3′ of STP A>G**						
**G/G**	1.00	1.00	1.00	1.00	1.00	1.00
**G/A**	1.21 (0.71,2.07)	1.16 (0.51,2.63)	0.75 (0.32,1.78)	1.60 (0.67,3.84)	1.39 (0.65,3.00)	0.58 (0.17,1.92)
**A/A**	1.16 (0.66,2.03)	1.14 (0.47,2.75)	1.01 (0.39,2.62)	2.54 (0.96,6.72)	1.12 (0.50,2.54)	0.58 (0.16,2.10)
**P_ trend_**		0.816	0.778	0.053	0.906	0.438
**ESR2 5696 bp 3′ of STP A>G**						
**A/A**	1.00	1.00	1.00	1.00	1.00	1.00
**A/G,G/G**	1.04 (0.61,1.75)	1.21 (0.58,2.55)	0.51 (0.19,1.37)	1.2 (0.51,2.85)	1.07 (0.57,2.02)	0.82 (0.22,3.06)
**P_ trend_**		0.613	0.183	0.676	0.835	0.773
**ESR2 5772 bp 3′ of STP A>G**						
**A/A**	1.00	1.00	1.00	1.00	1.00	1.00
**A/G,G/G**	1.07 (0.64,1.79)	0.63 (0.23,1.72)	1.23 (0.59,2.56)	1.03 (0.43,2.50)	1.19 (0.61,2.33)	0.98 (0.26,3.67)
**P_ trend_**		0.369	0.589	0.943	0.611	0.974

*Phytoestrogen: low (n = 254), high (n = 245); ‘Low’ and ‘High’ is defined as below and above the median total phytoestrogen intake of controls, 2.71 mg/mo. Missing values were excluded from the analysis.

† BMI: <25 (n = 222 ), 25–30 (n = 387), ≥30 (n = 100 ).

‡ NA: no applicable estimate due to small sample number.

All odds ratios are adjusted for age and race.

Overall, there was little evidence to suggest an interaction between genotypes and BMI, except that obese men (BMI≥30 kg/m^2^) with heterozygote G/A genotype in ESR2 38 bp 3′ of STP G>A had a 72% lower risk of prostate cancer (P = 0.026), and that more A alleles in ESR2 5659 bp 3′ of STP G>A were associated with increased prostate cancer risk in men with BMI <25 kg/m^2^ (p-value for trend = 0.053). Similar results were observed when BMI of 27 or 27 kg/m^2^ was used as the cutoff for grouping (data not shown).

In men with a T/T genotype in ESR1 Ex1+392T>C, A/A genotype in ESR1 Ex8+229G>A, and A/G or G/G genotype in ESR2 5696 bp 3′ of STP A>G (Table 4), high phytoestrogen group had a 58% (P = 0.048), 64% (P = 0.047) and 80% (P = 0.034) lower risk of developing prostate cancer, respectively. In contrast, in men with G/C, C/C genotype in ESR1 Ex1-192G>C, the high phytoestrogen group had 3.3 times the odds of developing prostate cancer compared with the low phytoestrogen group (P = 0.034).

**Table 4:** The effect of phytoestrogen* and BMI on the risk of prostate cancer by the genotypes of estrogen receptor gene SNPs.

SNP	Odds Ratio (95%CI)					
(case/control)	Phytoestrogen*		BMI†			
	Low (101/153)	High (93/152)	<25 (89/133)	25–30 (141/246)	≥30 (39/61)	P_ trend_
**Overall**	1.00	0.86 (0.55,1.33)	1.00	0.84 (0.59,1.19)	0.95 (0.57,1.57)	
**ESR1 Ex4-122C>G**						
**C/C**	1.00	0.89 (0.51,1.55)	1.00	**0.55 (0.34,0.89)**	0.57 (0.28,1.15)	**0.036**
**C/G**	1.00	0.85 (0.42,1.71)	1.00	1.15 (0.61,2.17)	1.52 (0.56,4.12)	0.426
**G/G**	1.00	0.91 (0.06,14.34)	1.00	0.39 (0.05,3.03)	0.43 (0.06,2.96)	0.393
**ESR1 Ex1+392T>C**						
**T/T**	1.00	**0.42 (0.18,0.99)**	1.00	0.65 (0.32,1.33)	1.10 (0.41,2.93)	0.851
**T/C, C/C**	1.00	1.23 (0.74,2.04)	1.00	**0.63 (0.41,0.98)**	0.56 (0.28,1.08)	**0.036**
**ESR1 Ex1-192G>C**						
**G/G**	1.00	0.64 (0.39,1.02)	1.00	**0.60 (0.40,0.91)**	0.82 (0.45,1.47)	0.166
**G/C,C/C** *	1.00	**3.30 (1.09,9.96)**	1.00	0.98 (0.38,2.54)	0.79 (0.19,3.24)	0.781
**ESR1 IVS6+52G>T**						
**G/G**	1.00	0.82 (0.51,1.32)	1.00	0.70 (0.46,1.07)	0.93 (0.51,1.72)	0.431
**G/T**	1.00	1.38 (0.53,3.55)	1.00	**0.39 (0.16,0.95)**	0.36 (0.11,1.17)	**0.059**
**T/T**	1.00	‡NA	1.00	2.62 (0.11,64.84)	‡NA	0.864
**ESR1 -104062C>T**						
**C/C**	1.00	0.79 (0.47,1.33)	1.00	**0.59 (0.37,0.93)**	0.85 (0.43,1.67)	0.237
**C/T, T/T**	1.00	0.91 (0.38,2.19)	1.00	0.86 (0.41,1.79)	0.50 (0.16,1.56)	0.271
**ESR1 Ex8+229G>A**						
**G/G**	1.00	1.99 (0.96,4.13)	1.00	0.58 (0.31,1.09)	0.93 (0.39,2.25)	0.479
**G/A**	1.00	0.87 (0.47,1.63)	1.00	0.82 (0.47,1.42)	0.68 (0.31,1.50)	0.318
**A/A**	1.00	**0.34 (0.12,0.97)**	1.00	0.58 (0.23,1.43)	0.50 (0.12,2.06)	0.231
**ESR2 38 bp 3′ of STP G>A**						
**G/G**	1.00	0.84 (0.42,1.69)	1.00	0.79 (0.42,1.50)	1.33 (0.58,3.00)	0.654
**G/A, A/A**	1.00	0.87 (0.51,1.49)	1.00	0.64 (0.40,1.02)	**0.43 (0.20,0.93)**	**0.015**
**ESR2 5659 bp 3′ of STP A>G**						
**G/G**	1.00	0.95 (0.30,3.03)	1.00	0.98 (0.34,12.82)	2.22 (0.63,7.79)	0.269
**G/A, A/A**	1.00	0.78 (0.48,1.25)	1.00	**0.65 (0.43,0.97)**	0.63 (0.33,1.19)	0.054
**ESR2 5696 bp 3′ of STP A>G**						
**A/A**	1.00	1.08 (0.69,1.71)	1.00	0.72 (0.48,1.08)	0.83 (0.47,1.48)	0.310
**A/G,G/G**	1.00	**0.20 (0.05,0.89)**	1.00	0.56 (0.20,1.57)	0.44 (0.10,1.98)	0.227
**ESR2 5772 bp 3′ of STP A>G**						
**A/A**	1.00	0.83 (0.53,1.30)	1.00	**0.66 (0.44,0.99)**	0.73 (0.41,1.30)	0.125
**A/G,G/G**	1.00	0.47 (0.40,5.44)	1.00	0.71 (0.25,2.02)	0.83 (0.20,3.41)	0.703

*For phytoestrogen, additional adjustment for total energy intake was made; all odds ratios are adjusted for age and race.

† Low and High is defined as below and above the median total phytoestrogen intake of controls.

‡ NA: no applicable estimate due to small sample number.

A higher BMI was not associated with prostate cancer risk [OR (95% CI) = 0.84 (0.59, 1.19) in over-weight men, and OR (95% CI) = 0.95 (0.57, 1.57) in obese men]. In men with certain SNPs, a trend in reductions in prostate cancer was noted with increased BMI. In particular, for men with G/A, G/G genotype in ESR2 38 bp 3′ of STP G>A, having a BMI of ≥30 kg/m^2^ was associated with a reduced the odds of developing prostate cancer by 57% compared to having a BMI of <25 kg/m^2^ (p-value for trend = 0.01) (Table 4).

An interaction association by BMI on the relationship between prostate cancer risk and ESR genotypes was suggested for ESR2 38 bp 3′ of STP G>A (P = 0.031). Interaction association by intake level of phytoestrogen on the relationship between prostate cancer risk and ESR genotypes was suggested for both ESR1 Ex1-192G>C (P = 0.0009) and ESR1 Ex8+229G>A (P = 0.044).

## Discussion

In this study, there was no overall association between prostate cancer risk and genotypic and allelic frequencies of ESR1 and ESR2 SNPs. Among those who were diagnosed with advanced prostate cancer, associations between prostate cancer risk and genotypes were suggestive for four SNPs: ESR1 Ex1+392T>C, ESR1 IVS6+52G>T, ESR2 38 bp 3′ of STP G>A and ESR2 5659 bp 3′ of STP A>G. Exploratory analyses suggested potential interactions between environmental exposure (BMI/phytoestrogen), and polymorphic variations in estrogen receptor genes resulting in differential prostate cancer risks.

With respect to ESR1, eight studies have addressed the same question as the present study did. In a Japanese study, codon 10(T→C) was associated with a 2-fold increased risk for prostate cancer (OR = 2.03, 95% CI: 1.17–3.53) [22]. Another study, also in Japan, reported a significant association of the T/T genotype of the PvuII site in the ESR1 (OR = 3.44; 95% CI:1.97–5.99) [23]. This finding was confirmed by an UK study (OR = 4.65; 95% CI:1.60–13.49) [24] and an Indian study (OR = 2.15, 95% CI:1.06–4.37) [25]. In a study in the U.S., a possible association was found between prostate cancer risk and ESR1 intronic restriction site, XbaI and PuII, but the association was not statistically significant. [26]. Another study found an association between the AG genotype, as well as presence of the G allele within the XbaI ESR1 SNP and prostate cancer risk, but no association between the *Pvu*II SNP and prostate cancer in black men [27]. In a French study, variant of the GGGA polymorphism from the ESR1 was associated with an increased risk of developing prostate cancer [28], [29]. Recently, Cunningham et al. have reported null association between ESR1 SNPs: IVS1-397, g34288C/T (rs2234693), IVS1-351, g3433A/G (rs9340799), ESR1 TA repeat polymorphism and prostate risk [30]. Conversely, McIntyre et al. observed that prostate cancer risk was greater with ESR1 (TA)_24_ and (TA)_25_ carriers [31]. However, none of the SNPs mentioned above overlapped with the SNPs examined in this study.

Consistent with our study findings, Cancel-Tassin et al. (2003) reported no association between prostate cancer risk and genotypes of ESR1 Ex1+392T>C and ESR2 Ex8+229G>A [28]. In that study, ESR1 Ex4-122C>G was shown to be associated with breast cancer [41] and the progression of prostate cancer [42], which is commensurate with our findings of higher risk for being diagnosed to have advanced disease. However, the authors did not find an association with prostate cancer incidence. In addition, Medeiros et al. (2003) reported a link of ESR1 Ex4-122C>G to unfavorable outcome parameters such as high pathologic grade and clinical stage [42], a finding consistent with ours that clinical stage was associated with the genotypes of ESR1 Ex4-122C>G (p value from chi-square test = 0.05).

Four previous studies have been published regarding association between polymorphisms in ESR2 and prostate cancer risk. One study was conducted in China, and the genotype and allele frequency of rs3829768 (A/G) and rs1271572 (C/A) in the upstream region of proximal promoter was significantly lower in prostate cancer cases than controls (P<0.01) [32]. The other study was conducted in Sweden with findings that genotype and allele frequency of rs2987983 (T/C) in the promoter region was associated with prostate cancer risk [33], and that the protective effect of phytoestrogen on prostate cancer was significant among men with carriers homozygous for the wild type allele (TT) of the same SNP [34]. Of recent, two studies have reported null association between ESR2 CA repeat polymorphism and prostate cancer [30], [31]. In a French study, additional 14 ESR2 SNPs were noted to have no association with prostate cancer risk [29]. Consistent with our findings, Cunningham et al. [30] observed null association between ESR2 3′togene, g.49888G/A (rs4986938). Except for this one study [30], previous studies have not reported on the SNPs included in the present study. For example, while a study in Sweden investigated four SNPs in the promoter region and introns of ESR2, the SNPs examined in this study were in the downstream non-coding region of ESR2 [33], [34].

A few epidemiological studies supported the hypothesis of a protective association between phytoestrogen (isoflavone) intake and prostate and breast cancers [20], [21]. Our study, however, did not show overall protective association of phytoestrogen intake for prostate cancer but did find a suggested interaction with two ESR1 SNPs (rs746432, rs2228480). Swedish study has identified rs2987983 in the promoter region of ESR2, which was not included in our study, as a potential effect modifier in the relationship between the intake of phytoestrogen and the risk of prostate cancer [34]. Two aspects of the data on phytoestrogen intake should be noted. First, missing data may have compromised the validity for assessing the association between phytoestrogen consumption and prostate cancer risk. In CLUE II, the food frequency questionnaire did not include soy beans or soy products such as soy milk and tofu. However, these are not expected to be a predominant source of phytoestrogens in this population. Furthermore, at the time of the CLUE study enrollment in 1989, soy products were not prevalent in the American diet. This study estimated the amount of phytoestrogen (isoflavone) intake by summing up the intakes of three legume items (beans, peas, peanuts), which were the predominant sources of phytoestrogen in the American diet. In summing the intakes of legume items, since missing one item led to missing data on the sum of all items, the proportion of missing data went up to around 30%. A high proportion of missing data significantly decreases the sample size available for statistical analysis, and consequently decreases statistical power. However, in imputation analysis, inserting zero or median in place of missing data did not affect the overall results. Second, the amount of phytoestrogen consumed in the Washington County was much less than that in Southeast Asia where soy is consumed habitually in moderate to large quantity. This may be one of the reasons that explained the discrepancies found between studies in Asia and the present study [21].

With regard to the test for interactive effects, the significance level (type I error rate) is the probability of falsely reporting significant interaction. Assuming the same effects across strata, the probability of finding at least one significant interaction by chance alone when undertaking 20 independent subgroup analyses such as in table 4 is 65% [43]. When the corrected p value for over-inflated false positive rate [44], 0.0025 (0.05÷20), is applied to table 4, one interaction remains statistically significant: with ESR1 Ex1-192G>C (log likelihood ratio test: p = 0.0009<0.0025), suggesting that this SNP was a strong effect modifier on the association between dietary intake of phytoestrogen and prostate cancer risk.

A major limitation of this study is that only a subset of known SNPs in two genes, ESR1 and ESR2, were examined:. Only 3 out of 10 selected SNPs (ESR1 Ex1+392T>C, ESR2 Ex8+229G>A, and ESR1 Ex4-122C>G) were studied in the past [28], [42], where null associations with prostate cancer risk were observed, consistent with our study findings. However, for the other 7 SNPs selected, our group was the first to report no overall association between those SNPs and prostate cancer incidence.

Functions of all candidate SNPs remain unclear. All of the four ESR1 SNPs in exons were synonymous polymorphisms with no associated amino acid change. Therefore, it is unlikely that these polymorphisms are causative. Yet, they may be in linkage disequilibrium with an unknown causative variant. Or, they can cause a structural change in RNA, altering translation efficacy, and thereby, leading to a change in ESR1 gene expression rate [22]. The situation is the same for other SNPs either in non-coding regions or in introns, warranting further functional or expression studies.

Tests of prostate-specific antigen (PSA) have been increasingly used for screening and diagnosis of prostate cancer since early 1990s. Differential use of PSA test between cases and controls may result in detection bias. In this study, there was no evidence of over-diagnoses of early cancer using PSA tests and digital rectal examinations (DRE) [44]. No appreciable difference in PSA test rate between cases and controls was observed. We had few African Americans and no Asians in the cohort, so we did not examine the associations in various ethnic groups.

In summary, no overall statistically significant association between prostate cancer risk and the selected ten SNPs in ESR1 and ESR2 genes was observed. However, four SNPs (rs2077647, rs2273206, rs4986938, rs928554) may be linked with higher risk for being diagnosed to have advanced stage disease. In addition, there may be an interactive effect between BMI/phytoestrogen and ESR genotypes on the risk of prostate cancer. Further investigations are needed to see if the study is replicable in other populations, especially in other ethnic groups, and to find out how the gene-environment interaction can be explained under the biological models.

## Supporting Information

Table S1 Hardy Weinberg Equilibrium (HWE) among Caucasian cases and controls
(0.02 MB XLS)

Table S2 Observed estrogen receptor SNP frequency in CLUE controls and SNP500/dbSNP data by race
(0.02 MB XLS)
